# Manganese Homeostasis in Cyanobacteria

**DOI:** 10.3390/plants9010018

**Published:** 2019-12-23

**Authors:** Marion Eisenhut

**Affiliations:** Biochemie der Pflanzen, Heinrich-Heine-Universität Düsseldorf, 40225 Düsseldorf, Germany; m.eisenhut@uni-duesseldorf.de

**Keywords:** manganese, cyanobacteria, homeostasis, transporter, photosystem II, Mn cluster, regulation

## Abstract

Manganese (Mn) is essential for life on earth. As a catalyst of the water oxidation reaction within photosystem II, the trace metal is responsible for the evolution of virtually all oxygen in the earth’s atmosphere. Mn acts furthermore as an activator or cofactor of numerous enzymes involved in reactive oxygen species scavenging or central and secondary metabolism. While the sufficient supply of oxygenic photosynthetic organisms with Mn is obvious for maintaining photosynthetic activity, the avoidance of cellular Mn overload is also critical. In this review, current knowledge about the Mn homeostasis network in the model cyanobacterium *Synechocystis* sp. PCC 6803 is presented, including transporters and regulators.

## 1. Introduction—The Essential Function of Manganese in Cyanobacteria

Manganese (Mn) is the 12th most abundant element in the earth’s crust [1] but only occurring in the nM range in the aquatic environment [2,3,4]. The transition metal is an essential micronutrient to all organisms since it activates or acts as a redox-active cofactor of central enzymes, such as Mn-dependent superoxide dismutase, glycosyl transferases, pyruvate carboxylase, ligninase, or oxalate oxidase [5,6]. Its favorable biochemical properties are reviewed in detail by Schmidt and Husted [6] within this special issue. Cyanobacteria evolved more than three billion years ago and were the first to use the oxidative power of Mn for running oxygenic photosynthesis [7]. Recent studies show that anoxygenic photosynthetic microorganisms also use Mn^2+^-ions as electron donors for phototrophy. It is possible that this light-dependent anaerobic microbial Mn oxidation process had already evolved during the Archaean eon, before the evolution of oxygenic photosynthesis [8,9]. Coordinated in a cluster of four Mn, one Ca, and five oxygen ions (Mn_4_CaO_5_), the Mn cluster as part of the oxygen-evolving complex (OEC) in photosystem II (PSII) catalyzes the oxidation of H_2_O to extract electrons for the re-reduction of the central chlorophyll P680, which has donated its electron to the photosynthetic electron transfer chain upon excitation by light absorption [10,11]. Additional proton products and molecular oxygen are also generated (reviewed in [12]). The ability to perform oxygenic photosynthesis is strictly bound to the occurrence of a Mn cluster [13]. Another metal replacing Mn has not been found yet. Thus, it is of little surprise that photosynthetic activity immediately drops upon Mn deprivation in both cyanobacteria [14] and plants [15,16].

Driving oxygenic photosynthesis obviously implies the careful management of Mn homeostasis. On the one hand, sufficient provision of Mn to PSII and other Mn-dependent enzymes needs to be guaranteed. Oxygenic photosynthetic organisms, such as cyanobacteria, contain 100-times more Mn on a cellular basis than non-photosynthetic organisms [17]. On the other hand, the overaccumulation of Mn needs to be avoided since this may lead to mismetallation of for example, typically Mg- or Fe-binding metalloenzymes, thus changing or inhibiting their activity [18,19].

## 2. The Manganese Homeostasis Network in *Synechocystis*

**Figure 1:**
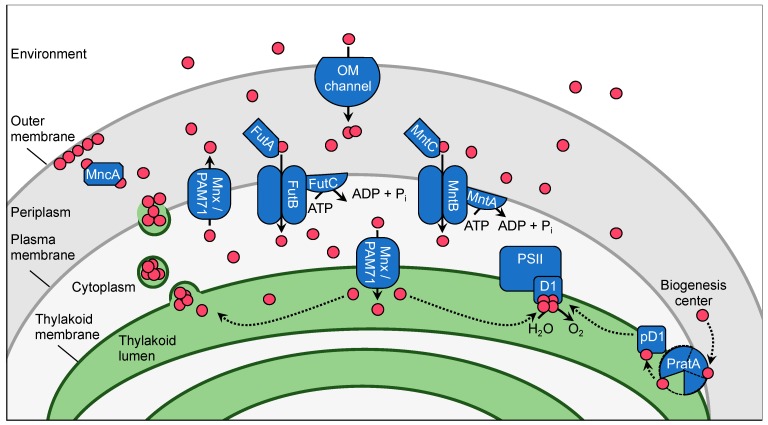
The Mn homeostasis network model for *Synechocystis*. Shown are proteins for which functions contributing to Mn homeostasis were assigned. Mn^2+^-ions are displayed as pink-colored circles. For simplification, the two-component regulator system ManS/ManR was omitted and has been presented separately in Figure 2. Mn is primarily taken up from the aquatic environment in its Mn^2+^ ionic form via a light-dependent transport process through outer membrane (OM) channels. It accumulates in the periplasmic storage, either associated with the outer membrane or bound by Mn cupin A (MncA). From the periplasm Mn is imported into the cytoplasm on demand. The ABC-type transporter MntCAB serves the Mn import under Mn-limiting conditions, while FutABC is suggested to fulfil this function in a constitutive manner. In biogenesis centers PratA preloads pre-D1 with Mn^2+^-ions from the periplasmic storage. Alternatively, thylakoid membrane located Mnx/PAM71 assists in lumenal Mn supply to PSII. Mn is excreted into the periplasm by Mnx/PAM71 or possibly vesicle formation from the thylakoid system.

To avoid critical imbalances, Mn needs to be specifically and timely allocated to the place where it is needed and sequestered in a safe storage place if accumulating in excess. That is, key to Mn homeostasis management is the controlled uptake from the environment and appropriate intracellular distribution of the metal. Proteins known to function in the Mn homeostasis network in *Synechocystis* sp. PCC 6803 (hereafter *Synechocystis*) are presented in Figure 1. Names, gene IDs, and assigned functions are summarized in Table 1.

### 2.1. Subcellular Manganese Allocation

Cyanobacteria are gram-negative bacteria with an outer and an inner membrane surrounding the cell as an envelope. The periplasmic space in between these membranes serves as Mn storage. Experiments with the outer membrane permeable metal chelator EDTA revealed that *Synechocystis* cells contained two distinct Mn pools. One pool with about 75% of total cellular Mn was found in the periplasm and was released upon treatment with EDTA. The remaining 25% of total cellular Mn, that was approximately 1.5 × 10^6^ atoms cell^−1^ [17], could not be released from the cell by washing with EDTA. This pool was localized accordingly to the inside of the cell, the cytoplasm and thylakoid system [17]. Mn^2+^-ions of the periplasmic pool may accumulate in the range of 100 mM and are either mostly attached to the outer membrane [17] or bound to the Mn-binding protein Mn cupin A (MncA; Figure 1) [20]. The metal is recruited from here into the cytoplasmic pool upon demand e.g., for incorporation into Mn-dependent metalloenzymes, such as phosphoenolpyruvate carboxykinase or pyruvate carboxylase [5]. The greatest Mn demand is clearly from the Mn cluster, which is situated at the thylakoid lumenal side. The cyanobacteria-specific PratA-dependent delivery of the Mn cluster to the D1 protein during PSII biogenesis [21] is one option and described later in detail. Additionally, Mn^2+^-ions are likely to be supplied directly by uptake into the thylakoid lumen. The periplasm also serves as storage for conditionally excess Mn in case the cytoplasmic Mn concentration exceeds its physiological threshold. The thylakoid lumen is suggested to function as a second safe storage for sequestration of surplus Mn [22,23].

The high importance of maintaining the Mn homeostasis with its specific spatial pools in a very narrow beneficial window is indicated by physiological analyses in cyanobacteria but also other bacteria, such as *Streptococcus pneumoniae.* These studies demonstrate that an only three to five fold increased intracellular Mn concentration results in strong to complete growth inhibition [22,23,24].

### 2.2. Manganese Transporter

Transport proteins are the major regulatory distributors. Mn is predominantly taken up in its Mn^2+^ oxidation state and also shuttled in this form within the cell [5]. The metal first needs to cross the outer membrane and second traverse the plasma membrane into the cytoplasmic pool. To reach the thylakoid lumen a third transport step is required.

#### 2.2.1. Uptake at the Outer Membrane

Generally, it is assumed that the outer membrane of gram-negative bacteria is permeable to most small solutes due to the presence of outer membrane channel proteins with low selectivity [35]. Keren et al. [17] demonstrated that the uptake of Mn from the environment into the periplasm was not simply due to diffusion but happened in a membrane potential-dependent process. That is, Mn accumulation in the periplasm was light-driven and was inhibited in experiments upon treatment with the PSII acceptor-side inhibitor DCMU. Treatment with CCCP, which uncouples membrane potentials by functioning as a protonophore, resulted in release of the total periplasmic Mn pool. These inhibitor studies suggest that Mn uptake at the outer envelope and the retention of Mn storage in the periplasm rely on photosynthetic electron transport [17]. The mechanisms by which the light-dependent uptake at the outer membrane is accomplished is unclear. It can be assumed that the photosynthetic electron flow generates a proton gradient across the thylakoid membrane first. This membrane potential is subsequently transduced to the plasma membrane by unknown processes [36] and is here used by the Ton system to finally activate an outer membrane channel for Mn uptake from the environment. The Ton system is typically employed by gram-negative bacteria, such as *Escherichia coli* [37]. It was shown that the plasma membrane proteins ExbB/ExbD functioned as a transmitter of the energy harvested from the plasma membrane potential to the TonB protein, which facilitated the opening of an outer membrane channel. In this respect, it is interesting to mention that the expression of *exbB/exbD* and *tonB,* which are principally involved in iron acquisition [38], was found to be enhanced by the deletion of the Mn-sensing two component system ManS/ManR in *Synechocystis*, which mimics Mn limitation [39]. It is possible that a TonB/ExbB/ExbD complex is also involved in activating outer membrane channels for Mn uptake in *Synechocystis.* However, this hypothesis has so far been purely speculative and needs to be experimentally tested. Mutant analysis has proven to be difficult, since the *Synechocystis* genome contains three *exbB-exbD* operons [38], and at least four genes encode outer membrane channel proteins [40].

#### 2.2.2. Uptake at the Plasma Membrane

Uptake of solutes at the plasma membrane is a selective process, facilitated by transport proteins with either a rather wide or highly specific substrate range. In cyanobacteria, at least two different systems serve Mn import from the periplasm into the cytoplasm. On the molecular level, the ATP-binding cassette (ABC) -type Mn transporter MntCAB [28,29] was identified as a Mn uptake system. With the kinetic parameters, K_m_ = 1–3 µM and V_max_ = 3–8 pmol min^−1^ 10^8^ cells^−1^, MntCAB is characterized as a high-affinity Mn transporter [29]. It comprises the typical subunits of an ABC-type transporter, with MntC serving as periplasmic substrate binding protein, MntA functioning as cytoplasmic ATP-binding subunit, and the integral membrane protein MntB facilitating the permease as the transport function [28]. Importantly, expression of the *mntCAB* operon is negatively regulated by the ManS/ManR two-component system [31,32] and only in place under Mn-limiting conditions [29,30]. This makes a second Mn import system mandatory, which serves to maintain Mn supply under sufficient Mn conditions. The assumption is supported by the observation that a mutant in *mntCAB*, which shows retarded growth under Mn-limiting conditions, can be compensated by addition of MnCl_2_ in the µM range [29]. As a candidate, FutABC is postulated to function in constitutive Mn uptake at the plasma membrane [30]. FutABC is an ABC-type transporter like MntCAB but typically uses iron (Fe) as a substrate. It is likely that this transporter accepts also Mn with lower affinity and imports it into the cytoplasm in a piggybacking mode [22,30].

#### 2.2.3. Uptake at the Thylakoid Membrane

Only very recently a protein facilitating Mn transport from the cytoplasm into the thylakoid lumen was identified. The protein Mn exporter (Mnx; [22]), also known as photosynthesis affected mutant 71 (PAM71; [23]), belongs to the unknown protein family (UPF) 0016. UPF0016 proteins are highly conserved in oxygenic photoautotrophs, pointing to a transport substrate with a special function in oxygenic photosynthesis. In the model plant *Arabidopsis thaliana*, the two homologs chloroplast manganese transporter 1 (CMT1) and PAM71 were shown to be indispensable for Mn provision to the chloroplast. Loss of the envelope localized CMT1 or thylakoid residing PAM71 resulted in strongly reduced loading of PSII with Mn. Obviously, the major substrate of both CMT1 and PAM71 is Mn [41,42]. Mn as the preferred substrate was also demonstrated for the homologous protein Mnx in *Synechocystis*. The knockout mutant ∆*mnx/pam71* was highly sensitive toward treatment with elevated Mn levels in the medium [22,23]. Further analyses revealed that the mutant accumulates Mn inside the cell, shows reduced Mn export activity, is impaired in PSII activity, and mounts a typical Mn intoxication phenotype with reduced chlorophyll *a* and PSI accumulation [22,23]. Obviously, Mnx/PAM71 is involved in export of Mn into the periplasm. However, how this sequestration is executed is unclear. First it needs to be clarified in which membrane Mnx/PAM71 resides. According to microscopic analysis using a Mnx fluorescence protein fusion variant that replaces the native endogenous Mnx, Mnx/PAM71 resides in the thylakoid membrane [22]. Immunoblot analysis of sucrose gradient fractions from ∆*pam71* cells expressing p*am71* in fusion with a His-tag under the control of the *petJ* promoter indicates Mnx/PAM71 is predominantly located in the plasma and only to a lesser amount in the thylakoid membrane [23]. Plasma membrane located Mnx/PAM71 can simply export Mn from the cytoplasm into the periplasmic space [23], while the export from the thylakoid lumen is more difficult to explain. It is feasible that either i) *Synechocystis* uses its biogenesis centers, which are at the interface between plasma and thylakoid membrane and thus connect periplasm and thylakoid lumen, for the exchange of Mn, or ii) the cyanobacterium possibly excretes Mn from the thylakoid lumen by vesicle formation [22].

The transport mode of Mnx/PAM71 and other members of the UPF0016 is unknown. For PAM71 in *Arabidopsis thaliana* is was hypothesized that the uptake of Mn into the thylakoid lumen worked in antiport with protons [42]. Though this hypothetical transport mode would work well at the thylakoid membrane, it would not be applicable to the UPF0016 transporters acting at the plasma membrane or the plastid inner envelope, since the proton gradient is not steep enough [43]. Future studies on localization, kinetic parameters, substrate range, and transport mode will aid in characterizing the function of Mnx/PAM71 in the Mn homeostasis network in more detail.

### 2.3. Manganese Delivery to Mn Cluster in PSII

The delivery and incorporation of Mn to the OEC at the lumenal side of the thylakoid membrane system in the cyanobacterial cell is largely unclear. So far, only the processing associated tetratricopeptide repeat protein A (PratA) could be assigned to function in this process [25,26,27], yet the functional mechanism remains to be clarified. The protein resides at the interface between the plasma and thylakoid membrane and is suggested to play a role in Mn incorporation into the D1 precursor (pD1) in the so-called biogenesis centers [27] (Figure 1). In yeast two-hybrid and pull-down assays, interaction between PratA and pD1 or mature D1 was shown [25,26,27]. More precisely, PratA binds to the C terminus of mature D1 protein, in close proximity to the Mn cluster [27]. Stengel et al. [27] demonstrated furthermore that PratA is a Mn^2+^-metalloprotein, which contains a high-affinity Mn^2+^-binding site (*K*_d_ = 73 µM). A knock-out mutant in *pratA* is strongly impaired in oxygen evolution [25], likely since delivery of Mn to the OEC in PSII is affected [27]. However, the mutant is able to perform photoautotrophic growth with strongly reduced rates [25]. This result implies that a PratA-independent, alternative option for Mn delivery to the OEC must exist in *Synechocystis*. A possible candidate for assistant delivery of Mn to PSII is the thylakoid transporter Mnx/PAM71, as discussed above.

Besides the Mn provision and its assembly as an active cluster, the turnover of the Mn cluster during repair of PSII has also been enigmatic. Due to the production of reactive oxygen species, light constantly damages the D1 reaction center and makes repair, that is replacement of the D1 protein, inevitable. The process of PSII repair is well studied (reviewed in e.g., [21,44]). In short, PSII disassembles into monomers and D1 is degraded by FtsH2/H3 proteases. Newly synthesized D1 is inserted into PSII, PSII reassembles as a dimer, and the Mn cluster is mounted and photoactivated. The fate of the Mn cluster during the repair process is unclear. It might either stay in the thylakoid lumen and get recycled, or the Mn might be “single use” and needs to be replaced by freshly imported Mn. A mutant in *mnx/pam71* reacted susceptible to high light treatment with prolonged recovery time [22]. This result can be interpreted as an indication for Mnx/PAM71 to facilitate Mn import into the thylakoid lumen, which is especially critical for Mn supply during on-site repair of PSII. Alternatively, the high-light sensitive phenotype of the mutant might also be interpreted as a result of Mn-induced inhibition of D1 biosynthesis [22]. Further studies are needed to clarify the role in Mnx/PAM71 in PSII repair.

## 3. Manganese Limitation—Perception and Response

**Figure 2:**
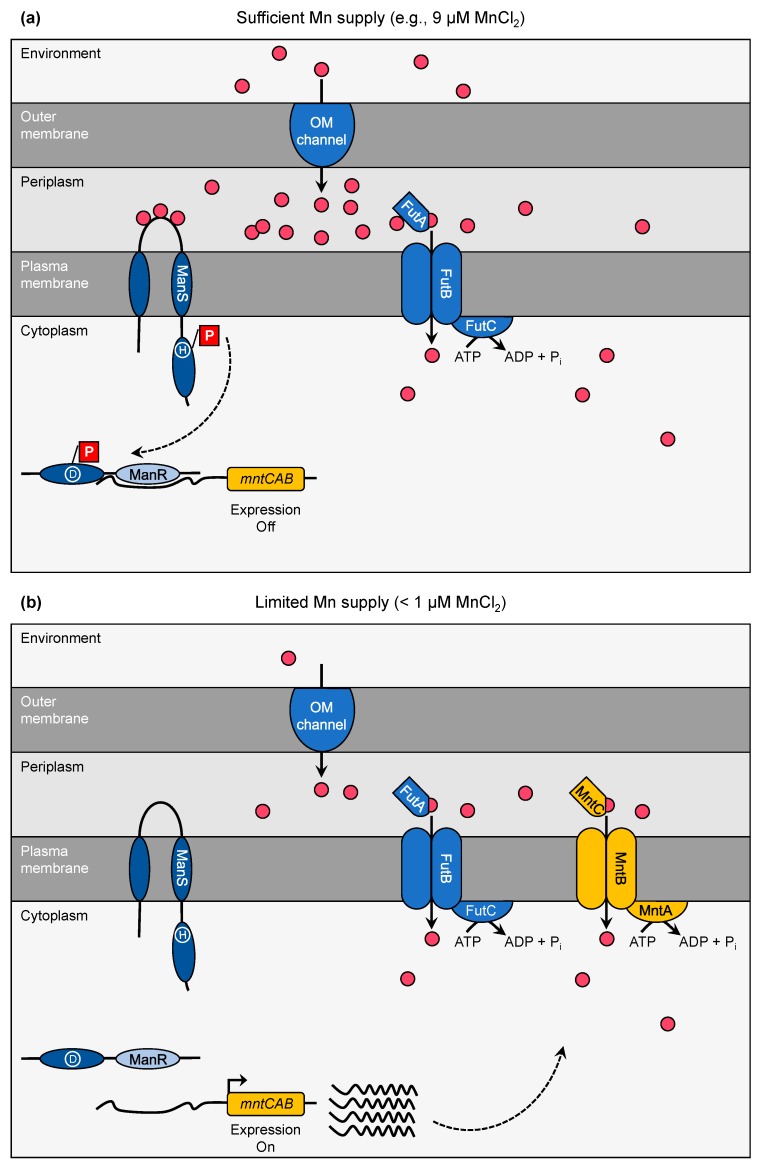
Model for the Mn-dependent operating mode of the ManS/ManR two-component system in *Synechocystis*. (**a**) At sufficient Mn supply (e.g., 9 µM MnCl_2_) the sensor protein ManS binds Mn^2+^-ions (pink-colored circles) in its periplasmic loop domain. This leads to phosphorylation (depicted as red box) of a His residue (encircled H) in its kinase domain. Thus, the activated sensor phosphorylates an Asp residue (encircled D) in the receiver domain of the response regulator ManR, resulting in binding of the DNA-binding domain (light-blue) to the promoter of the *mntCAB* operon (orange), thus blocking expression. (**b**) Under Mn-limiting conditions (<1 µM MnCl_2_) ManS is inactive and does not phosphorylate ManR. As a result, expression of the *mntCAB* operon takes place and leads to assembly of the MntCAB at the plasma membrane to maintain sufficient Mn import.

Regular BG11 medium used for cultivation of *Synechocystis* cells in the laboratory contains 9 µM MnCl_2_ [45]. In contrast, in natural aquatic habitats, such as oceans or lakes, Mn levels are in the 0.1–10 nM range [2,3,4] with soluble Mn^2+^-ions as the predominant form. Due to likely bacteria-mediated oxidation processes insoluble Mn^3+^/Mn^4+^-oxides are formed and the element can become restricting [4,46]. Though Mn is generally not considered to be a limiting factor for cyanobacteria, Mn concentrations < 100 nM affect the PS apparatus and reduce PSII activity, i.e., oxygen evolution capacity [14]. To prevent the lack of Mn provision to the cell and ensure maintenance of cellular functionality, *Synechocystis* employs a two-component signal transduction pathway operated by ManS and ManR (Figure 2).

So far, only the ManS/ManR system has been reliably identified to be involved in transcriptional regulation of Mn homeostasis in cyanobacteria. The manganese sensing (ManS) protein and the manganese response regulator (ManR) constitute a two-component system [31,32]. ManS functions as the critical sensor for Mn availability. As long as sufficient Mn (e.g., 9 µM MnCl_2_ as contained in regular BG 11 medium, [45]) is available (Figure 2a), ManS binds Mn^2+^-ions at the periplasmic loop region, which connects the two transmembrane domains of the protein. Upon Mn^2+^-binding, a His residue in the His kinase domain gets phosphorylated. Thus, the activated sensor protein transmits phosphorylation of an Asp residue in the receiver domain of ManR. In consequence, phosphorylated ManR binds to the promoter region of the target DNA and blocks expression (negative regulator). In case Mn concentrations in the environment become limiting (<1 µM, [31], Figure 2b), ManS does not associate with Mn^2+^-ions, stays inactive and accordingly does not phosphorylate ManR. Due to inactive ManR, expression of the target genes is no longer blocked and leads to the biosynthesis of the desired proteins. Microarray studies of knock-out mutants revealed that the *mntCAB* operon was the specific regulatory target of the ManS/ManR system [31,32]. However, a more recent RT-qPCR analysis showed that ManS/ManR also affected the transcript abundances of *futABC, exbBD*, and *tonA* [39]. It needs to be clarified whether expression of these genes is also directly controlled by binding of ManR to the promoter region as demonstrated for the *mntCAB* operon [31] or a secondary effect caused by alterations in global metal homeostasis.

In addition to the ManS/ManR system, the repeated five-residue domain A (RfrA) protein was postulated to function in the regulation of Mn uptake in *Synechocystis*. Though a mutant in *rfrA* showed reduced high-affinity Mn uptake activity [33], the nature of the direct target, a hypothetical second high-affinity Mn importer, and the regulatory mechanism remains to be clarified.

## 4. Manganese Excess—Risks and Avoidance

In contrast to Mn limitation, the Mn excess situation possibly resulting from high levels in the environment, is relatively unexplored in cyanobacteria. Physiological Mn intoxication symptoms range from reduced chlorophyll *a* biosynthesis and photosynthetic activity to cell death in *Synechocystis* [22,23,39]. These symptoms can be explained with mismetallation of metalloenzymes involved in these processes. Metals compete with other metals for binding to amino acid residues of active metal sites in accordance with the Irving–Williams series [47]: Mg^2+^ < Mn^2+^ < Fe^2+^ < Co^2+^ < Ni^2+^ < Cu^2^+ > Zn^2+^. Depending on recruitment of the site with the correct (metallation) or incorrect (mismetallation) metal cofactor, proteins become functionally activated or inactivated. In vivo, correct metallation is favored since the content of metals is specifically controlled in the cytoplasm [19]. Well-studied examples in this respect are the periplasmic cupins MncA (Mn-binding) and CucA (Cu-binding) in *Synechocystis* [20]. In the case of critical overaccumulation of Mn in the cytoplasm, mostly metalloenzymes typically binding Mg^2+^ or Fe^2+^ undergo mismetallation. It was observed that high Mn treatment led to accumulation of the chlorophyll *a* intermediate Mg-protoporphyrin IX in *Anacystis nidulans* [48]. Obviously, the intermediate is not converted further due to inhibition of one of the following enzymes in the biosynthesis pathway. Most probably, the diiron enzyme Mg-protoporphyrin IX monomethyl ester cyclase [49,50] is inactivated due to mismetallation, that is the incorporation of Mn instead of Fe. As a consequence, a chlorotic phenotype as Mn intoxication symptom is mounted.

A Mn toxicity avoidance strategy is the sequestration of surplus Mn into subcompartments, where the risk of mismetallation is low. The periplasm and thylakoid lumen are suggested to function as those safe places. The transporter protein Mnx/PAM71 seems to play an essential role in the prevention of cytoplasmic Mn overaccumulation [22,23] as discussed above.

## Figures and Tables

**Table 1:** List of proteins assigned to function in the Mn homeostasis network in *Synechocystis*.

Protein	Gene ID ^1^	Assigned Function	Reference
MncA	*sll*1358	Periplasmic Mn metalloprotein	[20]
PratA	*slr*2048	Delivery of Mn to pD1 protein during PSII biosynthesis	[25,26,27]
MntC	*sll*1598	High-affinity Mn uptake at the plasma membrane under Mn-limiting conditions	[28,29]
MntA	*sll*1599		
MntB	*sll*1600		
FutA1	*slr*1295	Candidate for constitutive Mn uptake at the plasma membrane	[30]
FutA2	*slr*0513		
FutB	*slr*0327		
FutC	*sll*1878		
Mnx/PAM71	*sll*0615	Mn export from cytoplasm into thylakoid lumen and periplasm, respectively	[22,23]
ManS/Hik27	*slr*0640	Mn-sensing two-component system to control expression of the *mntCAB* operon	[31,32]
ManR/Rre16	*slr*1837		
RfrA	*sll*1350	Expression regulator of unknown high-affinity Mn importer at the plasma membrane	[33]

^1^ acc. to Cyanobase [34].
